# Calorie restriction with regular chow, but not a high-fat diet, delays onset of spontaneous osteoarthritis in the Hartley guinea pig model

**DOI:** 10.1186/s13075-019-1925-8

**Published:** 2019-06-13

**Authors:** Lauren B. Radakovich, Angela J. Marolf, Lauren A. Culver, Kelly S. Santangelo

**Affiliations:** 1 Department of Microbiology, Immunology, Pathology, Colorado State University, 200 West Lake Street, Fort Collins, CO 80521 USA; 2 Department of Environmental and Radiological Health Sciences, Colorado State University, 123 Flint Cancer Center, Fort Collins, CO 80523 USA

**Keywords:** Osteoarthritis, High-fat diet, Obesity, Calorie restriction, Hartley guinea pig

## Abstract

**Background:**

Obesity is a leading risk factor for osteoarthritis (OA). In contrast, calorie restriction (CR) may lessen OA due to improved systemic inflammatory status and reduced weight-bearing. The aim of this study was to determine how CR with regular chow versus a high-fat diet (HFD) alters OA progression using the Hartley guinea pig model of disease.

**Methods:**

Twenty-four male guinea pigs were allocated to four groups at 2 months of age: (1) ad libitum regular chow (obese), (2) CR regular chow (lean), (3) ad libitum HFD, and (4) CR HFD. Animals in both HFD groups ate identical amounts and were combined into one HFD group for analyses. At 5 months, hind limbs were harvested for microcomputed tomography (microCT) and histopathologic evaluation of knee OA. Total body, gonad fat, and infrapatellar fat pad (IFP) masses were recorded. IFPs were collected for gene expression analysis. Immunohistochemistry for monocyte chemoattractant protein-1 (MCP-1) was performed on intact joints. Serum was utilized for protein C3 measurement. All data were compared using ordinary one-way ANOVA analyses with Tukey’s post-hoc tests.

**Results:**

Body mass in the lean and HFD groups were similar and lower than the obese group. Despite this, gonad fat pads in the HFD group were comparable to the obese group. MicroCT and histologic OA scores were similar in obese and HFD groups; both scores were significantly lower in the lean group. Obese and HFD groups displayed increased gene expression of pro-inflammatory and catabolic mediators in IFPs relative to lean animals. Consistent with this, immunohistochemistry for MCP-1 in knee joints demonstrated strong positive staining in obese and HFD groups but was minimally detected in lean animals. Serum protein C3 levels were also statistically higher.

**Conclusions:**

This study demonstrated that CR with a regular chow diet lessened knee OA in the Hartley guinea pig and was associated with decreased local and systemic inflammation compared to obese animals. HFD animals, although under CR conditions, had OA scores and inflammatory markers similar to obese animals. Thus, diet composition, and not solely body weight, may be a key factor in development of OA.

**Electronic supplementary material:**

The online version of this article (10.1186/s13075-019-1925-8) contains supplementary material, which is available to authorized users.

## Background

Osteoarthritis (OA), particularly knee OA, is a debilitating degenerative disorder affecting many people worldwide [1]. While OA secondary to a joint injury is typically associated with a defined onset, naturally occurring OA occurs in the absence of specific trauma for reasons that are not yet understood. Advancing age has long been the primary risk factor for development of spontaneous OA [2]; however, obesity is encroaching on age as a leading OA hazard given the alarming rise in obesity rates across the globe [3, 4]. The relationship between OA and obesity has been an area of increasing interest to researchers, but the mechanisms connecting these two conditions remain loosely defined. Early studies suggested that increased mechanical loading on joints contributed to worsened OA in obese patients [5]. However, higher incidence of OA in non-weight-bearing joints, such as in the hands [6], indicates that increased joint load may not be the only factor at play. Thus, dietary factors, including food composition and total calorie intake, likely play a complex role in the development of primary OA.

Recent studies have placed emphasis on the role systemic metabolic disturbances may play in the development of OA [5, 7–10]. Obesity is often associated with chronic, low-grade systemic inflammation [11–14]. For example, as the volume of adipose tissue expands, it outgrows its blood supply, leading to tissue hypoxia and stimulation of pro-inflammatory cytokines [15]. Enhanced lipopolysaccharide translocation from the gut to the bloodstream is another contributor to a chronic inflammatory state in obese individuals, particularly in response to high dietary fat content [16]. Similarly, high levels of circulating saturated fatty acids stimulate innate immune receptors such as toll-like receptor 4, resulting in a heightened pro-inflammatory state [15]. Increased circulating leptin concentration in obese individuals can also promote inflammation via stimulation of cytokines such as interleukin (IL)-1 beta (IL-1β), tumor necrosis factor (TNF), and IL-6 [17]. Additionally, high leptin levels have been shown to promote OA via increased activity of cartilage-degrading matrix metalloproteinases (MMPs) and aggrecanases [15, 18].

Previous work examining the role of obesity in promoting OA has concentrated on utilizing high-fat diets (HFDs) in mouse [19–23], rat [16, 24], and, less often, rabbit [25] animal models. Many of these studies have focused on post-traumatic OA secondary to surgical injury with a lesser number centered on naturally-occurring OA. Collectively, these studies provide convincing evidence that high-fat diet-induced obesity leads to worsened OA severity. Many have demonstrated that it is the increased fat mass, not overall body weight, associated with high-fat diets that is correlated with OA severity [16, 19, 20, 22, 25]. This is likely attributed to the metabolically active, inflammatory cytokine-producing nature of this adipose tissue. Diets used in these rodent studies vary in the amount of fat present, although 40–60% fat (derived from animal-based saturated fats) is typical. While such a high percentage of fat is effective in inducing obesity in mice and rats, it does not accurately reflect human diets, which are closer to 30% fat in a standard Western diet [26].

While HFDs have a detrimental effect on health, calorie restriction has emerged as a lifestyle factor that can prolong life and decrease the likelihood of developing chronic diseases such as atherosclerosis, type II diabetes, and neurodegenerative disease [27]. These positive effects are due to decreased oxidant damage, increased DNA repair, and decreased production of inflammatory cytokines secondary to less adipose tissue mass [28–31]. Few studies, however, have examined the effects of calorie restriction on development of OA. In dogs, calorie restriction appeared to delay onset of OA [32, 33]. A long-term study in mice did not show any differences in histologic OA severity between ad libitum and calorie-restricted mice, although it should be noted that fat mass was not different between the groups at the end of the study [27]. There is also one study examining calorie restriction in the Hartley guinea pig model of spontaneous OA. This work demonstrated that calorie restriction resulted in lessened OA severity at both 9 and 18 months of age [34], time points that correspond to late- and end-stage disease, respectively. Given this, we were interested in pursuing the influence of calorie restriction at earlier ages in the time course of disease.

Guinea pigs are perhaps an underutilized rodent model of OA. In particular, the Hartley strain is a useful model of spontaneous knee joint OA, as it develops disease in a condensed time frame and demonstrates bilateral pathologic lesions that are nearly identical to those seen in humans with aging-related OA [35, 36]. Guinea pigs have also emerged as being suitable models for a variety of metabolic diseases, including cardiovascular disease, atherosclerosis, and type II diabetes [37–39]. Furthermore, guinea pig lipid metabolism is more comparable to human lipid metabolism than any other rodent [37, 40–42]. High-fat diets have been successfully used in guinea pigs to drive metabolic diseases, such as type II diabetes [37] and non-alcoholic fatty liver disease [43]. In contrast to mouse and rat studies using a high-fat diet, the diets used in these guinea pig studies tend to range from 20 to 30% fat, which is more typical of a Westernized human diet. High-fat diets used in guinea pig studies also have a mixture of saturated animal-based fats and unsaturated plant-based fats. As guinea pigs are more fastidious about diet than mice and rats, they do not tend to develop overt obesity in concert with metabolic derangements when fed high-fat diets [37, 43]. This may allow for evaluation of the metabolic effects of HFDs without the potential confounding influence of mechanical factors associated with overt obesity. To date, there are no studies exploring the potential role that a high-fat diet may play in the development of spontaneous OA in a guinea pig model.

Collectively, the aims of the current study were to compare and contrast the effects of overconsumption and calorie restriction using standard rodent chow to that of a HFD on the onset of spontaneous OA in a guinea pig model. We hypothesized that animals on a HFD would exhibit worsened OA scores compared to both ad libitum and calorie-restricted animals on a regular chow diet. Likewise, we expected calorie restriction to result in improved OA scores at this early time point.

## Methods

### Animals

All procedures were approved by the university’s Institutional Animal Care and Use Committee and were performed in accordance with the NIH Guide for the Care and Use of Laboratory Animals. Twenty-four 6- to 7-week-old male Dunkin-Hartley guinea pigs were purchased from a commercial vendor (Charles River Laboratories, Wilmington, MA). Animals were maintained at Colorado State University’s Laboratory Animal Resources housing facilities and were monitored daily by a veterinarian. All guinea pigs were singly-housed in solid bottom cages and provided ad libitum access to water daily.

After 1 week of acclimation, animals were randomly allocated to one of four feeding groups: (1) ad libitum regular chow (obese), (2) calorie-restricted regular chow (lean), (3) ad libitum HFD, and (4) calorie-restricted HFD. As laboratory-raised guinea pigs fed ad libitum often become obese with age [44], this first group was referred to as such in the current study. Further, as animals in group 3 were noted to self-restrict consumption of the HFD to the same amount as those in group 4 (25 g), this group was condensed to a single HFD group. All animals were harvested at 5 months of age, a time when this strain of guinea pig has recognized signs of early OA [35].

### Diet composition

The regular chow diet (Teklad Global Guinea Pig Diet #2040, Madison, WI) provided 31% calories from protein, 12% from fat, and 57% from carbohydrate. This diet was supplemented with vitamin C (1050 mg/kg). Fat was derived from linseed meal. Protein and carbohydrates were derived from a mixture of alfalfa, wheat, oats, and fish meal. Animals fed ad libitum ate between 50 and 60 g of chow daily. Animals on the restricted regular chow diet received 30 g of food daily for the duration of the study [27]. The HFD (#151006, Dyets Inc., Bethlehem, PA) provided 18% calories from protein, 30% from fat, and 52% from carbohydrates. Fat was sourced from a mixture of Primex vegetable shortening and beef tallow. The source of protein was isolated soy protein, and carbohydrates were derived from sucrose and fructose. All animals receiving the HFD consumed 25 g of food each day, which matched the calorie consumption of the restricted regular chow group.

### Tissue collection

At the time of harvest, animals were anesthetized with a mixture of isoflurane and oxygen. Body weights were recorded at this time. Thoracic cavities were opened, and blood was collected with 20-gauge butterfly catheter via direct cardiac puncture. After blood collection, anesthetized animals were immediately transferred to a carbon dioxide chamber for euthanasia. Hind limbs were removed at the coxofemoral joint. The left limb was placed into 10% neutral buffered formalin for 48 h and then transferred to PBS for microCT analysis. After microCT imaging was complete, tibial length was measured using calipers. Limbs were then transferred to a 12.5% solution of ethylenediaminetetraacetic acid (EDTA) at pH 7 for decalcification. EDTA was replaced twice weekly for 6 weeks.

After the right hind limb was removed, the knee joint was exposed by dissecting through the quadriceps muscles and reflecting the patella distally. The infrapatellar fat pad (IFP) was removed from the patellar tendon, weighed, and then placed in All Protect Tissue Reagent (Qiagen) for gene expression analysis. Fat from the left epididymis (hence forth referred to as gonad fat) was removed and weighed. A section of masseter muscle was collected from each animal, and its width was measured.

### MicroCT

Knee joints were scanned using the Inveon microPET/CT system (Siemens Medical Solutions, Malvern PA), with a voxel size of 18 μm, a voltage of 100 kV, and an exposure time of 2356 ms. Clinical features of OA were scored on reconstructed microCT images using a whole-joint grading scheme developed by our lab in conjunction with a veterinary radiologist [45]. Features graded include presence/size and location of osteophytes, subchondral bone cystic changes, subchondral bone sclerosis, articular bone lysis, and intraarticular soft tissue mineralization. Images were scored in duplicate in a random order, blinded to diet group. An intraclass correlation coefficient of 1.0 for intra-reviewer consistency was calculated.

### Histologic grading of OA

After decalcification, coronal slices of the knees at the level of the medial tibial plateau were sectioned, as previously described [35]. Samples were paraffin embedded and a 5-μm intact central section was stained with toluidine blue. Medial and lateral femoral condyles, along with medial and lateral tibial plateaus, were scored using the recommended published guidelines [35]. This semiquantitative histopathologic grading scheme is based on articular cartilage structure, proteoglycan content, cellularity, tidemark integrity, and presence of osteophytes. Scores were performed in a blinded fashion by two independent pathologists (LBR and KSS). Scores from each of the four anatomic locations were summed to obtain a total knee joint OA score for each guinea pig. An intraclass correlation coefficient of 0.9 for between reviewer consistency was calculated.

### Immunohistochemistry for MCP-1 on knee joints

Immunohistochemistry (IHC) was performed on sections of knee joints using a polyclonal rabbit antibody to monocyte chemoattractant protein-1 (MCP-1) (Abcam ab9669) at a dilution of 1:100. Prior to incubation with primary antibody, slides were incubated in citrate buffer overnight at 55 °C for antigen retrieval, as recommended for skeletal tissues [46]. Slides were incubated in primary antibody overnight at 4 °C, followed by a 30-min incubation with a biotinylated goat anti-rabbit secondary antibody. Bone marrow hematopoietic cells and macrophages frequently exhibited MCP-1 staining, serving as internal positive controls for each section. Exposure to secondary antibody, alone, or rabbit immunoglobulin at a concentration matching that of the primary antibody did not result in any positive immunostaining. Sections were counterstained with hematoxylin, coverslipped, and viewed by light microscopy. At least four sections from each joint were examined for immunostaining in chondrocytes and associated matrix. The mean percentage of positive cells and mean integrated intensity (calculated as pixel area times mean intensity) were determined in articular cartilage (superficial, middle, and deep zones) from both femoral condyles and tibial plateaus using Image-Pro® (Media Cybernetics, Rockville, MD). All calculations were performed using identical thresholds across all photographs.

### Complete blood count, serum biochemical profile and serum protein C3 measurement

At the time of harvest, blood collected via cardiac puncture was allocated to 0.5 mL EDTA microtubes for complete blood count (CBC). CBCs were performed at the CSU Clinical Pathology Laboratory using the Advia 120 hematology analyzer (Siemens, Munich, Germany) with instrument settings and software specifically designed for guinea pig samples. Blood films were manually reviewed, and a leukocyte differential count was performed by a veterinary clinical pathologist (LR). Remaining blood taken at euthanasia was placed in red top glass tubes to incite clotting. After 30 min, red top tubes were adequately clotted and placed into a centrifuge at 5000×*g* for 10 min for serum collection. One aliquot of serum was submitted to the Colorado State University Clinical Pathology Laboratory for serum biochemical analysis using the Roche Cobas 6000 (Basel, Switzerland). Remaining serum was aliquoted to cryovials, snap frozen in liquid nitrogen, and then stored at − 80 °C for C3 analysis. Serum protein C3 levels were measured on snap frozen serum using a guinea pig-specific ELISA (Abcam ab157705) according to the manufacturer’s protocol.

### Gene expression of IFP and gonad fat using NanoString technology

Total RNA was extracted from IFP and gonad fat samples using an RNeasy Lipid Tissue Mini Kit (Qiagen, Hilden, Germany). RNA was quantified spectrophotometrically with a NanoDrop™ 2000 (ThermoFisher Scientific, Waltham, MA). A total of 250 ng of RNA, at a concentration of 20 ng/μl, was sent to the University of Arizona Genetics Core for analysis. Custom, guinea pig-specific probes were designed and synthesized by NanoString Technologies (see Table 1 for genes analyzed). Data analysis was performed using nSolver™ software provided by NanoString Technologies. Results, reported as absolute transcript counts, were normalized to positive controls and housekeeping genes. Data for the IFP is provided in the manuscript proper. Additional file 1: Table S1 provides gene expression data for gonad fat among the groups; Additional file 2: Table S2 compares gene expression data for IFP versus gonad fat within the three groups.

**Table 1:** Normalized absolute nCounter mRNA counts present in the IFP. Data is represented as the mean (range)

Gene	Function	Obese	Lean	HFD	*P* value
IFNγ	Pro-inflammatory cytokine	7.222 (4.360–11.440)	15.64 (7.060–20.44)	16.31 (2.490–37.06)	0.1204
IL-10	Anti-inflammatory cytokine	11.95 (9.680–14.87)	8.283 (2.350–13.74)	13.94 (3.660–23.90)	0.1372
IL-1β	Pro-inflammatory cytokine	30.41 (15.75–71.81)	29.45 (24.01–39.37)	42.97 (22.21–53.90)	0.0944
IL-4	Th2 response, tissue repair/fibrosis	15.54 (13.73–20.83)	13.60 (7.060–17.58)	22.63 (13.13–37.33)	0.0112^*^
IL-5	Promotes Ig production and eosinophil activation	18.13 (12.91–22.65)	13.12 (9.750–18.09)	25.97 (16.04–41.05)	0.0029^*^
IL-6	Pro-inflammatory cytokine	25.01 (20.12–29.16)	29.60 (18.68–45.15)	58.82 (21.73–76.84)	0.0151^+^
LIF	Anti-inflammatory cytokine	23.78 (18.37–32.85)	28.21 (16.52–38.43)	28.71 (12.02–42.72)	0.5087
MCP-1	Pro-inflammatory cytokine	333.3 (265.4–487.0)	439.4 (361.0–510.5)	564.9 (398.7–812.7)	0.0005^+^
NFκB	Pro-inflammatory transcription factor	112.1 (95.20–132.7)	119.7 (102.8–145.1)	161.0 (107.8–231.4)	0.0015^*,+^
COX2	Pro-inflammatory enzyme	32.61 (23.52–54.11)	34.99 (29.61–48.63)	52.55 (35.37–69.86)	0.0010^*,+^
Tacr1	Binds Substance P, pro-inflammatory, causes pain	205.3 (142.0–276.1)	167.8 (121.6–254.9)	211.3 (107.4–340.4)	0.3172
TGF-β1	Tissue repair, both pro and anti-inflammatory	275.2 (195.1–403.5)	254.9 (206.1–330.4)	375.3 (210.6–591.1)	0.0173^*^
TNF	Pro-inflammatory cytokine	10.92 (8.710–13.53)	8.082 (7.060–9.000)	15.07 (7.290–22.49)	0.0009^*,+^
HIF1α	Responds to hypoxia	1640 (1116–2020)	1552 (893.8–2654)	1899 (1170–3089)	0.4487
MMP13	Cleaves type II collagen	19.74 (13.12–31.65)	11.88 (8.790–14.26)	35.43 (15.31–59.03)	0.0059^*,+^
MMP2	Cleaves type IV collagen	8192 (6133–10,346)	9722 (7571–12,566)	11,514 (8944–14,686)	0.0157^+^
MMP9	Cleaves type IV and V collagen, activates neutrophils	14.46 (2.610–46.53)	17.47 (8.250–23.08)	26.23 (2.250–64.05)	0.2951
Timp1	Inhibits MMPs	1310 (956.1–1709)	1076 (768.8–1563)	1145 (761.4–1826)	0.4103
Timp2	Inhibits MMPs	6270 (4494–7529)	7654 (6354–9109)	8014 (6089–10,405)	0.0248^+^
Adiponectin	Glucose sensitivity and FA oxidation	10,530 (8885–11,904)	20,830 (12666–24,246)	23,818 (9818–38,769)	0.0093^+^
Leptin	Inhibits hunger, resistance seen in obesity	6379 (3975–9012)	2296 (1191–3651)	9471 (3921–26,498)	0.0348^*^
LPL	Hydrolyzes TGs into FAs and glycerol	2843 (1188–3988)	16,118 (7900–22,842)	7536 (3271–16,678)	< 0.0001^*,+,†^
PPARγ	Lipid uptake and adipogenesis, insulin sensitization, anti-inflammatory	185.4 (105.7–249.1)	466.2 (275.3–736)	254.6 (139.2–426.2)	0.001^*,†^

*difference between HFD and lean groups; + difference between HFD and obese groups; † difference between obese and lean groups

### Statistical analyses

Group size and power were prospectively determined using the statistical software at www.stat.uiowa.edu/~rlenth/Power. Based on preliminary work, histologic assessment of OA was selected as the principle outcome. Utilizing a within-group error of 0.5 and a detectable contrast of 1.0 in a linear regression model, power associated with a Tukey/HSD post-test (alpha = 0.05) was calculated as 0.9 with a sample size of 6 per experimental group.

Data for total body weights, tibial length, gonad fat weight, IFP weight, masseter muscle width, histologic OA scores, clinical microCT OA scores, CBC values, serum biochemical values, serum protein C3 values, and NanoString mRNA normalized absolute counts were subjected to, and passed, normality testing via the Kolmogorov-Smirnov test. Data were compared using parametric ordinary one-way ANOVA analyses followed by Tukey’s multiple comparisons tests to allow for adjusted *P* values. Statistical significance was set at *P* < 0.05. All statistical analyses were performed with GraphPad Prism (La Jolla, CA, USA).

## Results

### General description of guinea pigs in each diet group

**Fig. 1:**
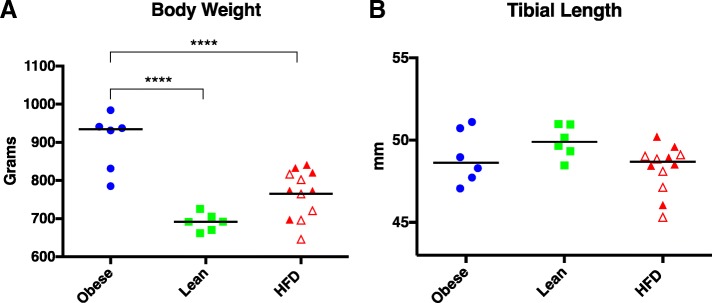
Total body weight (**a**) and tibial length (**b**) in obese, lean, and HFD groups. Black line represents mean value. Open red triangles designate animals receiving the restricted HFD; closed triangles define animals on the ad libitum HFD. ****P < 0.0001

All animals in each group survived the duration of the study. Final body weights for the obese group were significantly higher than both the lean and HFD groups. There was no difference in body weights between the lean and HFD groups (Fig. 1a). To ensure that differences in body weight were not attributable to variations in skeletal properties, tibial lengths from all animals were measured. There was no statistical difference in tibial length between any of the three diet groups (Fig. 1b). To determine if muscle mass may be different between the three groups, the width of the masseter muscle was measured and compared. Muscle width was significantly smaller in HFD group compared to the obese ad libitum regular chow group. Mean muscle width in the obese group was 5.8 mm (95% CI 4.4–7.2), 4.7 mm (95% CI 3.8–5.5) in the lean calorie-restricted group, and 3.6 mm (95% CI 3.1–4.1) in the HFD group (*P* = 0.0029).

**Fig. 2:**
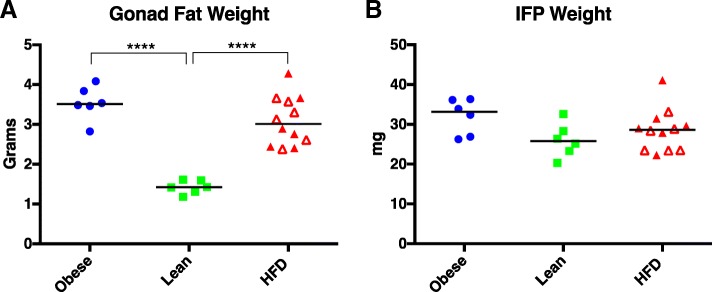
Weight of gonad fat (**a**) and IFP (**b**) in obese, lean, and HFD groups. Black line represents mean value. Open red triangles designate animals receiving the restricted HFD; closed triangles define animals on the ad libitum HFD. ****P < 0.0001

In addition to body weights, two adipose depots were also weighed and contrasted among the groups. Gonad fat (collected from the epididymis) was chosen to represent an abdominal adipose store, while the IFP was selected to represent a depot local to the knee joint. Despite differences in total body weights, gonad fat weights in the obese and HFD groups were similar. Gonad fat weight in both of these groups was significantly higher than that of the lean group (Fig. 2a). Interestingly, this pattern was not seen for IFP weight. IFP weights for all three groups were similar, with no statistical differences found (Fig. 2b).

### MicroCT and histologic assessment of OA

**Fig. 3:**
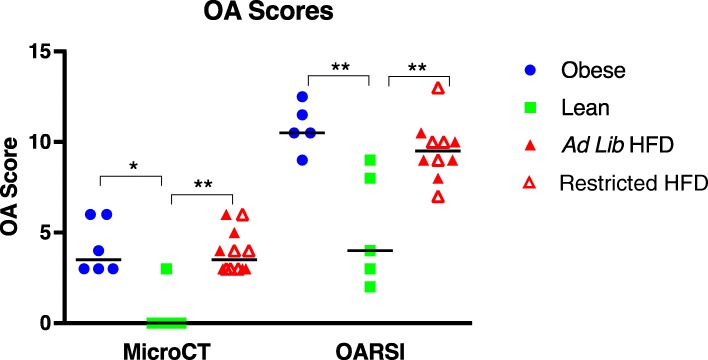
MicroCT and OARSI histologic scores in obese, lean, and HFD groups. Black line represents mean value. *P < 0.05, **P < 0.01

**Fig. 4:**
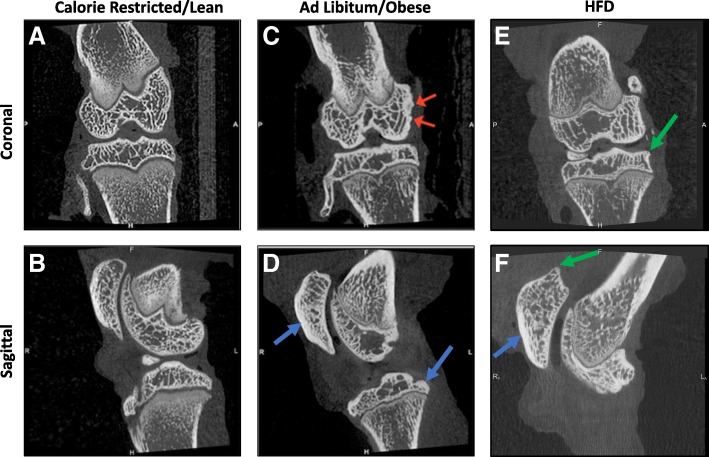
**a** Dorsal and **b** sagittal reconstructions from a calorie-restricted animal with no OA lesions. MicroCT OA score of 0. **c** Dorsal reconstruction from an obese animal. There is sclerosis and small enthesiophytes on the medial femoral condyle (red arrows) and mild sclerosis of the central tibial plateau. **d** Sagittal reconstruction from same animal. Mild sclerosis of the cranial patella and caudal tibial condyle is present (blue arrows). MicroCT OA score of 6. **e** Dorsal reconstruction of an animal on the HFD. On the medial tibial condyle, there is a small osteophyte (green arrow). **f** HFD sagittal reconstruction. There is a small osteophyte on the proximal patella (green arrow), as well as mild sclerosis on the cranial patella (blue arrow). MicroCT OA score of 4

Osteoarthritis was assessed via two methodologies. MicroCT was utilized to examine bony changes associated with OA, while histologic assessment of joints via the OARSI grading scheme was used to evaluate articular cartilage abnormalities. Using the clinical OA microCT scoring system, both obese and HFD groups had similar OA scores. Scores for these groups were statistically higher than those reported in the lean group (Fig. 3). Of note, in the lean group, many of the animals had no microCT evidence of OA at 5 months of age, a time when early bony changes are consistently present in Hartley guinea pigs [35]. All animals in both the obese and HFD group had visible small enthesiophytes and/or osteophytes present on the patella and/or the tibia (Fig. 4). Subcondral bone sclerosis was also seen on the cranial patella. In contrast, only one animal in the lean group had a small osteophyte present on the patella. All remaining lean animals were radiographically normal, with no bony changes associated with OA.

**Fig. 5:**
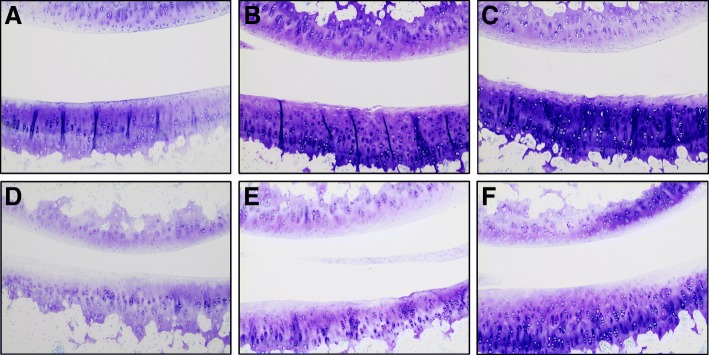
**a** T blue photomicrograph of the medial compartment from a calorie-restricted animal. The articular surface is smooth with only mild superficial proteoglycan loss. **b**, **c** T blue photomicrographs from obese animals. The articular surfaces are fibrillated, there is proteoglycan loss in the superficial and middle zones, and there is cell clustering. **d**–**f** T blue photomicrographs from HFD-fed animals. There is regional to diffuse proteoglycan loss in the superficial and middle zones, mild articular surface irregularity, occasional cell clustering, and focal cell loss within the superficial zone

The OARSI grading scheme resulted in a similar trend to that seen with the clinical microCT score. Again, both obese and HFD groups had similar OARSI OA scores that were significantly higher than those present in the lean group. Interestingly, although the total OA scores were similar in the obese and HFD groups, specific cartilage lesions appeared to differ. Articular cartilage surface fibrillation and superficial fissures were noted within the obese group (Fig. 5b, c). Superficial proteoglycan loss, with both focal and diffuse distributions, occasional chondrocyte clustering, and focal cell loss were noted in the HFD group (Fig. 5d–f). In the lean group, some animals exhibited mild superficial proteoglycan loss, but articular surface integrity was often maintained (Fig. 5a).

### CBC, serum biochemistry, and protein C3 data

**Fig. 6:**
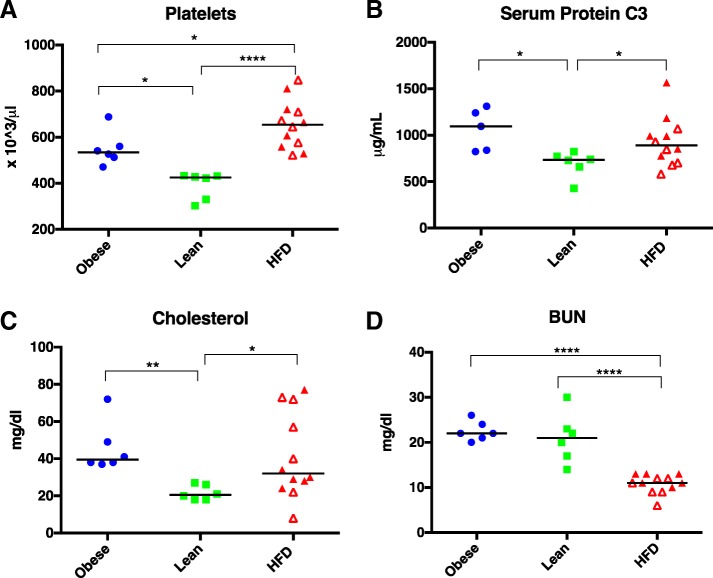
Total platelet counts (**a**), protein C3 levels (**b**), serum cholesterol (**c**), and serum BUN (**d**) in obese, lean, and HFD groups. Black line represents mean values. Open red triangles designate animals receiving the restricted HFD; closed triangles define animals on the ad libitum HFD. *P < 0.05, **P < 0.01, ****P < 0.0001

Complete blood count, serum biochemistry data, and serum protein C3 were assessed for evidence of systemic inflammation and other metabolic effects the diet conditions had on normal physiology. On CBC, platelet counts, which have been associated with increased levels of IL-6 in guinea pigs [47], were higher in the HFD group compared to both the lean and obese group. Additionally, platelet counts in the obese group were higher than those of the lean group, but not as high as the HFD group (Fig. 6a).

While increased platelet count can indicate a higher level of inflammation [48], it is non-specific and other indicators of inflammation, such as an increased white blood cell count, were not noted in this study. To more conclusively determine if a higher level of inflammation was present in the obese and HFD groups, a guinea pig-specific ELISA for protein C3, an acute phase reactant, was used to analyze serum. Serum protein C3 levels were similar among the obese and HFD groups and were significantly higher than levels present in the lean group (Fig. 6b).

In addition to the higher platelet and protein C3 levels, two other significant differences were noted on serum biochemical profiles. Total cholesterol levels were higher in both the obese and HFD groups compared to lean animals (Fig. 6c). Interestingly, there was a wide spread of cholesterol values for the HFD group, with some animals having values similar to those of the lean group and others having much higher serum concentrations. In addition, blood urea nitrogen (BUN), a protein breakdown product excreted by the kidneys, was similar in the obese and lean groups. BUN in the HFD group was significantly lower than levels seen in both the obese and lean groups (Fig. 6d).

### Gene expression data from the IFP

As the goal of this study was to determine how HFD and calorie restriction affect knee OA, we were particularly interested to see how these dietary manipulations might affect the IFP. Using NanoString technology, absolute mRNA counts for many pro- and anti-inflammatory cytokines, chemokines, and matrix metalloproteinase (MMP) genes were evaluated (Table 1). Numerous pro-inflammatory genes were upregulated in the HFD group. Compared to the lean group, the HFD animals had higher expression of several pro-inflammatory genes including IL-5, nuclear factor kappa beta (NFkB), cyclooxygenase 2 (COX2), transforming growth factor-β (TGF-β), and TNF. Compared to the obese group, HFD-fed animals exhibited higher expression of IL-6, MCP-1, NFkB, COX2, and TNF. Additionally, the HFD group had higher expression of matrix metalloproteinase-13 (MMP13), which cleaves type II collagen, than both the obese and lean groups. Expression of MMP2, which cleaves type IV collagen, and tissue inhibitor of metalloproteinases 2 (Timp2), an inhibitor of MMP activity, were also higher in the HFD group than the obese group.

When examining adipokines and other genes related to lipid metabolism, there were also several notable differences in gene expression among the groups. Leptin, an adipokine that normally inhibits hunger but exhibits resistance in obesity, was significantly increased in the HFD group compared to the lean group. It also trended towards being higher in the obese regular chow group compared to lean animals, but this did not reach statistical significance. Adiponectin, an adipokine that is traditionally decreased systemically in obesity, had higher gene expression in the IFP of the HFD group compared to the obese regular chow group. Lipoprotein lipase (LPL), which hydrolyzes triglycerides for cellular use, was increased in the lean regular chow group compared to both the obese regular chow and HFD groups. It was also higher in the HFD group compared to obese regular chow animals. Finally, peroxisome proliferator-activator gamma (PPARγ) was increased in the lean regular chow group compared to both the obese regular chow and HFD groups.

### Immunohistochemistry for MCP-1

**Fig. 7:**
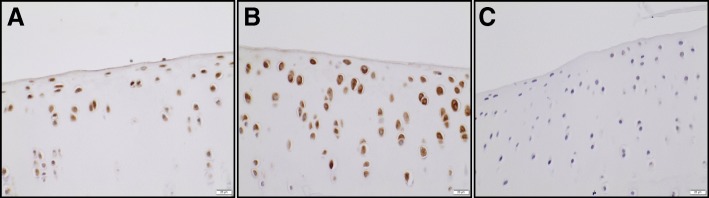
Representative images of immunostaining for MCP-1 on the medial tibial plateau for the ad libitum regular chow group (**a**), the HFD group (**b**), and calorie-restricted group (**c**). × 400

**Fig. 8:**
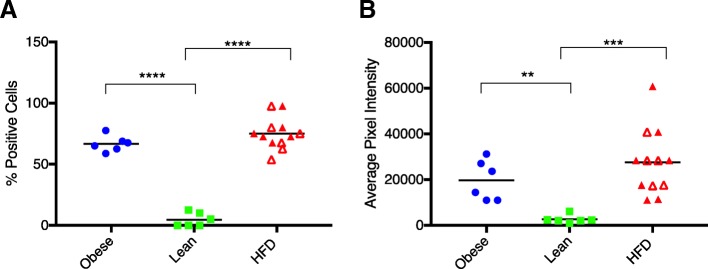
The mean percentage of positive cells (**a**) and mean integrated intensity (**b**) of MCP-1 immunostaining in articular cartilage from both femoral condyles and tibial plateaus in obese, lean, and HFD groups. Black line represents mean values. Open red triangles designate animals receiving the restricted HFD; closed triangles define animals on the ad libitum HFD. **P < 0.01, ***P < 0.001, ****P < 0.0001

Little to no MCP-1 immunostaining was seen within chondrocytes in the lean group (Fig. 7), which was confirmed by significantly lower mean percentage of positive cells and mean integrated intensity (Fig. 8). In contrast, MCP-1 protein expression was prominent within chondrocytes in the obese regular chow group and the HFD group, which is consistent with the microCT and histologic assessment of OA provided above. Staining for MCP-1 within the extracellular matrix was minimal in all three groups.

## Discussion

This study demonstrates that calorie-restriction using a low-fat, regular chow diet lessens OA severity early in disease onset in a guinea pig model of spontaneous disease. An interesting, yet unintended, outcome of the study was that animals fed the HFD essentially self-imposed calorie restriction, despite being fed ad libitum. Due to their fastidious nature, guinea pigs in this group ate less food than expected, resulting in body weights that were similar to those seen in the purposefully calorie-restricted, regular chow group. Despite similar body weights among the HFD and calorie-restricted groups, microCT and histologic OA scores were worse in the HFD group. Thus, the benefits of calorie-restriction appear to be diminished by the pro-inflammatory nature of the HFD. Our findings lend support to the growing body of evidence that the link between OA and adiposity may be attributable to systemic and local knee joint inflammation. Total body weight ranged from 645 to 816 g in the HFD group. Interestingly, heavier animals in this group had worse microCT OA scores (*r* = 0.62, *P* = 0.0313). Thus, increased weight-bearing on joints cannot be excluded as a factor in the development of knee joint OA. Rather, it seems likely that both loading forces and inflammation contribute to disease development.

In the current study, obese regular chow-fed animals had similar OA scores as leaner animals fed a HFD. Despite the difference in total body weight, a major abdominal fat depot was similarly sized between these two groups. These findings suggest that the HFD animals, although lean, had reduced lean muscle mass. This was corroborated by the decreased size of the masseter muscle in the HFD group compared to the obese regular chow group. The lower BUN in the HFD animals is another indication of lower muscle mass, but may also be attributed to decreased protein consumption in this group. When comparing the macronutrient composition of the regular chow and high-fat diets, the proportion of carbohydrates was similar; however, the HFD contained 30% fat and 18% protein, while the regular chow contained 12% fat and 31% protein. It is possible that either the lesser amount of protein and/or higher amount of fat content in the diet contributed to reduced muscle mass in the HFD group.

Regardless of body weight differences between the obese and HFD groups, they exhibited similar OA scores. This indicates that diet composition, and not simply body weight or calorie-restriction, alone, may play a key role in the development of primary OA. The abdominal fat depot was significantly smaller in the calorie-restricted regular chow group. This finding suggests that animals fed the HFD maintained higher levels of adiposity, with smaller muscle mass, despite having body weights comparable to the calorie-restricted regular chow group.

In contrast to the variably sized abdominal fat depot among the three diet groups, the local knee joint adipose tissue, the IFP, was similarly sized among all three diet groups. Thus, it appears that the IFP is well-preserved, even in the face of calorie restriction. While more exploration is required to define the specific role of the IFP in OA, the current findings suggest preservation of this tissue depot may be essential to joint health. A few studies employing HFD-induced mouse models of OA found that IFP volume or area increased after being fed a HFD [21, 23, 49]. Human studies examining relationships between body mass index (BMI) and IFP size have yielded conflicting results. At least two studies have found no association between body mass index and IFP size in both lean and overweight/obese individuals [50, 51]. Results of the current study mimic what has been reported in these human studies, with no differences in IFP weight found despite differing total body weights in guinea pigs. However, other studies have shown positive correlations between IFP size and body weight in women [52, 53]. More work is needed to further clarify the role of IFP in both healthy and obese individuals.

While overall mass of the IFP was similar among the three groups, gene expression profiles of the IFP differed. Previous studies have shown that IFPs removed from OA patients have increased gene expression of a multitude of cytokines [54, 55]. Thus, we were interested to see how dietary factors may affect IFP gene expression in the guinea pig model. In general, expression of pro-inflammatory genes was higher in the HFD group compared to both obese and calorie-restricted regular chow groups. Specifically, NFkB, a transcription factor, and downstream targets IL-6 and TNF exhibited increased expression in the HFD group. It is well-established that both HFDs and obesity incite a chronic, low-grade inflammatory state due to upregulated NFkB and subsequent secretion of inflammatory cytokines such as IL-1, IL-6, and TNF [56, 57]. Here, we demonstrate that upregulation of these genes occurs locally in the knee joint in the IFP, potentially directly contributing to inflammation and subsequent OA in guinea pigs fed a HFD. Other pro-inflammatory pathways that were upregulated in the HFD group include the COX2 pathway, as well as the chemotactic factor MCP-1. Many of these pro-inflammatory cytokines and adipokines are known to increase activity of cartilage-degrading MMPs [58].

The current study also demonstrated increased gene expression of MMP13 in the HFD group compared to both the obese ad libitum and lean calorie-restricted groups. MMP13 primarily degrades type II collagen, a major constituent of the extracellular cartilage matrix. Additionally, MMP2 gene expression was higher in the HFD group compared to the obese ad libitum group. MMP2 cleaves type IV collagen. Type IV collagen is found in the pericellular matrix directly adjacent to chondrocytes and has anti-angiogenic properties that may play a role in maintaining an avascular and hypoxic environment in normal, healthy cartilage [59]. In damaged cartilage, the relative abundance of type IV collagen drops precipitously [60]. Our data suggest that MMP activity originating from the IFP in obese and HFD-fed animals may contribute to degradation of types II and IV collagen in cartilage. Interestingly, while total OA scores were similar between the obese and HFD groups, the features contributing to these scores differed. Thus, it may be worthwhile to determine whether this alteration in MMP activity may explain the differences in OA pathology seen in these treatment groups.

Given the abundance of gene expression data indicating increased inflammation within knee joints of the HFD and obese regular chow groups, IHC probing for MCP-1 protein expression was performed. Expression of this protein in chondrocytes were similar between the obese regular chow and HFD groups, while expression was not present in the lean calorie-restricted group. This is in interesting contrast to the Nanostring data that indicated higher MCP-1 levels in the IFP of HFD animals compared to obese animals. The reason for this discrepancy is uncertain but is likely related to post-transcriptional regulation of MCP-1 [61] or differential expression of this protein in different tissue types. Regardless, these findings confirm that there is increased inflammation within the knee joint in the obese and HFD groups compared to the lean group. Likewise, this data supports the premise that calorie-restriction with a low-fat diet results in low levels of knee joint inflammation. MCP-1 is a pro-inflammatory chemokine that plays a key role in OA pathogenesis and neuropathic pain [62]. Synovial fluid MCP-1 levels have been associated with radiographic knee OA and clinical symptoms in humans [63, 64]. Human articular chondrocytes express MCP-1, which increases expression of MMPs resulting in proteoglycan loss in vitro [65, 66]. Additionally, administration of an MCP-1 signaling inhibitor protected cartilage after joint injury in a rat model [67].

In addition to evidence of local inflammation in the knee joint of the guinea pigs on the HFD, animals also had signs of increased systemic inflammation. While perhaps not at a level able to incite an increased white blood cell count on routine CBCs, the increased platelet count in both the HFD and obese regular chow groups compared to calorie-restricted regular chow animals may indicate a higher level of systemic inflammation. Cytokines, particularly IL-6, stimulate thrombopoiesis in the bone marrow [68], which results in a reactive thrombocytosis in a variety of inflammatory conditions, including obesity [69]. An increased platelet count, alone, however, is not specific for inflammation. Lack of differences in white blood cell numbers may be due to the low-grade or relatively short-term nature of the inflammatory response typically described with obesity. As such, we measured a more sensitive and specific biomarker of inflammation, serum protein C3, an acute phase reactant in guinea pigs [70]. This assay confirmed that both the HFD and obese regular chow groups had greater levels of circulating inflammatory proteins than CR regular chow animals. In humans, the major acute phase reactant, C-reactive protein, has been correlated to severity of radiographic OA [71]. Similar to these human studies, serum C3 levels were positively correlated to radiographic microCT OA score (*r* = 0.45, *P* = 0.0395) in the current study.

An intriguing finding of the current study was the variability in the types of cartilage lesions noted between the obese ad libitum group and the HFD group. Levels of systemic inflammation and overall OARSI OA scores for these two groups were similar; however, the types of lesions observed differed. In the obese ad libitum group, there were more fibrillations and fissures in the articular cartilage surface. In the HFD group, proteoglycan loss was more prominent. The reason for these differences should be pursued, although we speculate it may be due to locally driven changes and mechanical differences. Increased loading on joints in the obese group may more directly cause surface damage via formation of fissures. Loss of proteoglycan in the HFD group may be due to systemic inflammation or due to decreased protein content in the diet. The differences observed between the obese and HFD groups suggest local inflammation within the knee joint (including differences in IFP phenotype) are more influential to OA outcome than levels of systemic inflammation. Of note, types of lesions noted on microCT did not differ between these groups. More differences in bony changes may emerge if our analyses extended beyond 5 months of age.

Of note, the current study utilized only saturated fats in the HFD. This was necessitated in order to make a shelf-stable pellet at room temperature. However, it is well-established that saturated fats induce inflammation. Future investigations will alter fat composition of the diet to include unsaturated fats to determine if overall percentage of fat in the diet or fatty acid composition is more important in driving development of OA. It is also important to repeat this research using female animals to determine if there may be any sex differences in response to diet. Extending this study beyond 5 months of age is also needed to determine long-term effects of these diets. It would also be interesting to assess lameness via gait analysis or cage monitoring. Finally, directed mechanistic studies are necessary to identify specific pathways contributing to the pathology noted in this experiment. To the authors’ knowledge, this is the first study to examine the IFP in the guinea pig. Our results suggest the IFP plays a role in disease development and that inflammatory profiles of the IFP are altered by changes in diet. More work is warranted to further define the role of the IFP in the development of spontaneous OA in this model.

## Conclusions

This study provides several important conclusions regarding effects of diet on development of spontaneous OA. Firstly, calorie restriction was able to delay the onset of OA in disease-prone Hartley guinea pigs. Secondly, the benefit of calorie-restriction was diminished in animals eating a high-fat diet. This is likely due to the higher levels of systemic and local inflammation seen in these animals. Thus, diet composition, and not calorie content alone, must be considered a key player in development of OA. Additional studies are needed to determine if dietary composition influences OA outcomes in humans, but the current study indicates that high-fat diets may not be optimal for long-term knee joint health.

## Additional files

Additional file 1: **Table S1.** Normalized absolute nCounter mRNA counts present in gonad fat. Data is represented as the mean (range). (DOCX 16 kb)

Additional file 2: **Table S2.** Normalized absolute nCounter mRNA counts present in gonad fat versus IFP in the three groups. Data is represented as the mean (range). (DOCX 16 kb)

## Data Availability

The datasets used and analyzed during the current study are available from the corresponding author on reasonable request. All data generated or analyzed during this study are included in this published article [and its supplementary information files].

## Abbreviations

ANOVA: Analysis of variance
BMI: Body mass index
BUN: Blood urea nitrogen
CBC: Complete blood count
CI: Confidence interval
COX2: Cyclooxygenase 2
CSU: Colorado State University
DNA: Deoxyribonucleic acid
EDTA: Ethylenediaminetetraacetic acid
ELISA: Enzyme-linked immunosorbent assay
HFD: High-fat diet
IFP: Infrapatellar fat pad
IHC: Immunohistochemistry
IL: Interleukin
LPL: Lipoprotein lipase
MCP-1: Monocyte chemoattractant protein 1
MicroCT: Microcomputed tomography
MMP: Matrix metalloproteinase
mRNA: Messenger RNA
NFkB: Nuclear factor kappa B
NIH: National Institutes of Health
OA: Osteoarthritis
OARSI: Osteoarthritis Research Society International
PBS: Phosphate-buffered saline
PPARγ: Peroxisome proliferator-activator gamma
RNA: Ribonucleic acid
T blue: Toluidine blue
TGF-β: Transforming growth factor beta
Timp2: Tissue inhibitor of matrix metalloproteinase 2
TNF: Tumor necrosis factor
