# Supplementary material for: Calorie restriction with regular chow, but not a high-fat diet, delays onset of spontaneous osteoarthritis in the Hartley guinea pig model
Source: Arthritis Res Ther. 2019 Jun 13;21:145. doi: 10.1186/s13075-019-1925-8 (PMC6567638; doi:10.1186/s13075-019-1925-8)
Supplement: Supplementary file 1 — Table S1. Normalized absolute nCounter mRNA counts present in gonad fat. Data is represented as the mean (range). (DOCX 16 kb) [file 13075_2019_1925_MOESM1_ESM.docx]

Additional file 1: Table S1 Normalized absolute nCounter mRNA counts present in gonad fat. Data is represented as the mean (range).

| **Gene** | **Function** | **Obese** | **Lean** | **HFD** | **P-value** |
| --- | --- | --- | --- | --- | --- |
| **IFNγ** | Pro-inflammatory cytokine | 43.64 (11.95-77.41) | 82.05 (42.25-106.1) | 58.83 (27.58-85.16) | **0.0138^†^** |
| **IL-10** | Anti-inflammatory cytokine | 19.25 (11.95-30.97) | 27.93 (8.02-92.33) | 15.93 (9.75-26.04) | 0.3551 |
| **IL-1β** | Pro-inflammatory cytokine | 135 (68.44-208.4) | 122.7 (70.11-234.6) | 124.3 (73.78-263) | 0.9120 |
| **IL-4** | Th2 response, tissue repair/fibrosis | 96.98 (42.83-165.1) | 79.4 (38.02-224.6) | 82.27 (31.25-126.8) | 0.7811 |
| **IL-5** | Promotes Ig production and eosinophil activation | 69.71 (46.39-99.09) | 77.56 (25.17-292) | 59.03 (27.45-89.28) | 0.7795 |
| **IL-6** | Pro-inflammatory cytokine | 44.83 (21.73-66.35) | 14.4 (11.27-17.11) | 30.37 (11.87-78.13) | **0.0271^†^** |
| **LIF** | Anti-inflammatory cytokine | 49.52 (37.85-69.19) | 12.45 (8.45-16.18) | 22.98 (13.52-31.98) | **<0.0001^+,†^** |
| **MCP-1** | Pro-inflammatory cytokine | 1543 (749.6-3118) | 539.2 (327.2-850.9) | 845.6 (460.7-1546) | **0.0057^+,†^** |
| **NFκB** | Pro-inflammatory transcription factor | 314 (228.1-381.9) | 327.1 (247.6-450.6) | 273.9 (193.4-348.1) | 0.1124 |
| **COX2** | Pro-inflammatory enzyme | 424 (248-566.4) | 360.4 (264.6-583.9) | 376.5 (175.7-556.5) | 0.6501 |
| **Tacr1** | Binds Substance P, pro-inflammatory, causes pain | 189.6 (93.56-266.1) | 86.85 (78.7-96.18) | 116.8 (54.13-164.3) | **0.0010^+,†^** |
| **TGF-β1** | Tissue repair, both pro and anti-inflammatory | 336.5 (263.2-381.9) | 224.7 (162.7-356.8) | 235.9 (164.4-364.9) | **0.0056^+,†^** |
| **TNF** | Pro-inflammatory cytokine | 25.25 (11.95-34.06) | 12.5 (4.58-18.31) | 17.92 (12.67-22.26) | **0.0100^†^** |
| **HIF1α** | Responds to hypoxia | 2434 (2221-2661) | 2446 (2030-2868) | 2879 (2078-4333) | 0.1132 |
| **MMP13** | Cleaves type II collagen | 9.538 (5.43-13.42) | 12.03 (9.89-13.74) | 11.23 (4.09-32.99) | 0.7655 |
| **MMP2** | Cleaves type IV collagen | 11985 (8840-17279) | 7812 (4122-13064) | 7842 (5876-10597) | **0.0091^+,†^** |
| **MMP9** | Cleaves type IV and V collagen, activates neutrophils | 15.74 (10.86-21.68) | 5.504 (3.44-7.19) | 9.638 (5.28-16.2) | **0.0002^+,†^** |
| **Timp1** | Inhibits MMPs | 1198 (672.4-2370) | 725.8 (449.4-1157) | 875.6 (409.3-2138) | 0.2861 |
| **Timp2** | Inhibits MMPs | 19435 (7504-23741) | 13484 (10212-15381) | 14829 (3679-20503) | 0.1015 |
| **Adiponectin** | Glucose sensitivity and FA oxidation | 81195 (26492-12378) | 161778 (92161-197895) | 112453 (20763-166409) | **0.0101^†^** |
| **Leptin** | Inhibits hunger, resistance seen in obesity | 236136 (61546-424182) | 158826 (64779-264722) | 251045 (46527-367525) | 0.2327 |
| **LPL** | Hydrolyzes TGs into FAs and glycerol | 24814 (12593-39733) | 150929 (66545-196488) | 50756 (27758-87638) | **<0.0001^*,†^** |
| **PPARγ** | Lipid uptake and adipogenesis, insulin sensitization, anti-inflammatory | 1222 (502-1602) | 3011 (1894-4443) | 1426 (637.2-2292) | **<0.0001^*,†^** |

* Different between HFD and Lean groups; + Different between HFD and Obese groups; † Different between Obese and Lean groups
