# Supplementary material for: Calorie restriction with regular chow, but not a high-fat diet, delays onset of spontaneous osteoarthritis in the Hartley guinea pig model
Source: Arthritis Res Ther. 2019 Jun 13;21:145. doi: 10.1186/s13075-019-1925-8 (PMC6567638; doi:10.1186/s13075-019-1925-8)
Supplement: Supplementary file 2 — Table S2. Normalized absolute nCounter mRNA counts present in gonad fat versus IFP in the three groups. Data is represented as the mean (range). (DOCX 16 kb) [file 13075_2019_1925_MOESM2_ESM.docx]

Additional file 2: Table S2. Normalized absolute nCounter mRNA counts present in gonad fat versus IFP in the three groups. Data is represented as the mean (range).

| **Gene** | **Obese** | | | **Lean** | | | **HFD** | | |
| --- | --- | --- | --- | --- | --- | --- | --- | --- | --- |
|  | ***IFP*** | ***Gonad*** | ***P-value*** | ***IFP*** | ***Gonad*** | ***P-value*** | ***IFP*** | ***Gonad*** | ***P-value*** |
| **IFNγ** | 14.7 (8.87-23.29) | 34.16 (10.72-63.77) | **0.0409** | 20.85 (9.41-27.26) | 69.99 (24.01-84) | **0.0062** | 28.32 (4.33-64.36) | 45.12 (10.88-74.27) | 0.1129 |
| **IL-10** | 24.33 (19.71-30.28) | 15.28 (9.2-24.36) | **0.0176** | 10.53 (3.14-18.31) | 12.29 (6.68-20.84) | 0.7132 | 24.21 (6.35-41.51) | 11.16 (7.23-17.51) | **0.0036** |
| **IL-1β** | 61.91 (32.06-146.2) | 105 (59.79-135.5) | 0.1944 | 40.6 (32.01-52.48) | 81.24 (57.63-111.1) | **0.0274** | 68.39 (16.42-93.61) | 93.15 (55.83-174.8) | 0.0590 |
| **IL-4** | 31.64 (27.95-42.4) | 76.59 (38.41-136.1) | 0.0515 | 18.82 (9.41-23.44) | 42.03 (21.61-64.25) | 0.0678 | 39.3 (22.8-64.82) | 63.12 (10.31-108.1) | **0.0219** |
| **IL-5** | 36.91 (26.28-46.11) | 55.92 (30.16-77.95) | 0.0866 | 16.16 (13-18.31) | 28.45 (20.81-51.22) | 0.1263 | 45.1 (27.86-71.29) | 44.08 (17.18-76.14) | 0.8975 |
| **IL-6** | 80.13 (40.97-226.2) | 34.43 (18.98-54.66) | 0.1991 | 39.6 (24.9-60.2) | 11.84 (6.4-15.63) | **0.0211** | 96.38 (32.9-196.4) | 20.04 (9.64-33.65) | **0.0006** |
| **LIF** | 48.41 (37.4-66.88) | 49.8 (33.22-49.8) | 0.1610 | 40.73 (30.87-51.23) | 10.5 (4.8-15.63) | **0.0019** | 49.86 (20.9-74.2) | 20.32 (10.22-53.26) | **<0.0001** |
| **MCP-1** | 678.6 (540.4-991.5) | 1172 (654.8-2027) | 0.0557 | 571.9 (481.2-680.6) | 374.1 (321.2-474.4) | **0.0119** | 996.5 (692.3-1441) | 625.1 (374.1-1065) | **0.0009** |
| **NFκB** | 228.3 (193.8-270.2) | 247.2 (195.3-300.4) | 0.5132 | 159.5 (137-193.4) | 266.2 (221.7-312.4) | **0.0017** | 279.7 (187.2-401.9) | 210.2 (114.7-263.5) | **0.0003** |
| **COX2** | 66.38 (47.88-110.2) | 31.56 (13.29-49.57) | **0.0340** | 46.74 (39.47-64.83) | 22.83 (8.1-57.63) | 0.1178 | 91.25 (61.43-121.3) | 32.48 (6.8-156.1) | **0.0035** |
| **Tacr1** | 417.9 (289.2-562.2) | 152.7 (60.83-219.3) | **0.0007** | 200.4 (162.1-274.1) | 70.91 (47.22-86.67) | **0.0084** | 367 (186.4-591.2) | 87.93 (22.91-140.1) | **<0.0001** |
| **TGF-β1** | 560.2 (397.2-821.4) | 266.1 (216.8-332.3) | **0.0076** | 319.7 (274.8-390) | 157.1 (128.8-179.5) | **0.0013** | 651.8 (365.7-1027) | 222.5 (105.9-695-3) | **0.0001** |
| **TNF** | 22.23 (17.73-27.54) | 19.93 (9.35-26.79) | 0.4823 | 11.04 (9.41-11.04) | 9.9 (3.82-13.02) | 0.5884 | 25.8 (12.66-39.05) | 13.98 (8.41-19.72) | **0.0025** |
| **HIF1α** | 3339 (2272-4113) | 1929 (1693-2387) | **0.0085** | 1775 (1192-2650) | 1900 (1630-2232) | 0.6246 | 3298 (2032-5365) | 2044 (1043-2569) | **0.0006** |
| **MMP13** | 77.74 (26.72-64.44) | 7.523 (4.75-10.56) | **0.0114** | 154.3 (11.72-452.1) | 9.013 (6.4-11.45) | 0.2507 | 89.51 (22.6-397.3) | 7.353 (2.63-13.04) | **0.0139** |
| **MMP2** | 15906 (12047-21062) | 9572 (6482-15499) | **0.0427** | 12962 (10094-16753) | 5514 (3435-7425) | 0.0007 | 18018 (11774-25504) | 5590 (2835-7695) | **<0.0001** |
| **MMP9** | 29.43 (5.32-94.72) | 12.44 (8.18-17.05) | 0.2573 | 72.69 (11-222.7) | 4.463 (2.86-6.48) | 0.2715 | 45.54 (3.91-111.2) | 8.628 (3.64-22.91) | **0.0012** |
| **Timp1** | 2450 (1267-3480) | 922.8 (526.6-1541) | **0.0025** | 1435 (1025-2084) | 586.9 (405-897.4) | **0.0017** | 2000 (1322-3172) | 623.1 (334.7-1267) | **<0.0001** |
| **Timp2** | 12765 (9149-15327) | 15190 (6731-19559) | 0.3373 | 10205 (8471-12145) | 11390 (6457-14587) | 0.5212 | 13917 (10574-18070) | 11492 (1214-18210) | 0.1595 |
| **Adiponectin** | 19170 (7830-24235) | 63518 (23762-101974) | **0.0177** | 27770 (16887-32325) | 138565 (20298-165980) | **0.0029** | 36836 (13215-67328) | 88610 (6849-156656) | **0.0057** |
| **Leptin** | 11420 (3597-18348) | 185054 (55204-349460) | **0.0148** | 3061 (1588-4867) | 140521 (36820-251051) | **0.0090** | 15473 (4748-46018) | 195553 (15348-313421) | **<0.0001** |
| **LPL** | 5788 (2418-8188) | 19457 (10736-31982) | **0.0187** | 21489 (10533-30454) | 130462 (37824-192879) | **0.0037** | 13087 (5681-28964) | 38003 (17896-80492) | **0.0023** |
| **PPARγ** | 377.4 (215.2-507.2) | 954.8 (450.3-1260) | **0.0080** | 621.6 (367-981.3) | 2545 (1077-3551) | **0.0040** | 418.5 (158.9-740.1) | 1085 (210.2-1732) | **0.0018** |
